# Exogenous 24-epibrassinolide mitigates damage in grape seedlings under low-temperature stress

**DOI:** 10.3389/fpls.2025.1487680

**Published:** 2025-02-18

**Authors:** Fengxia Dong, Xinyu Li, Chang Liu, Boxiang Zhao, Yu Ma, Wei Ji

**Affiliations:** ^1^ College of Horticulture, Shanxi Agricultural University, Jinzhong, China; ^2^ College of Chemical Engineering, Huaqiao University, Quanzhou, China; ^3^ Department of Horticulture and Crop Science, College of Food, Agricultural, and Environmental Sciences, The Ohio State University, Columbus, OH, United States

**Keywords:** grape seedlings, 24-epibrassinolide (EBR), low-temperature stress, physicochemical properties, mitigation mechanism

## Abstract

Grapes are cultivated worldwide and have a high economic value as fruit trees. However, winter frost damage and spring cold damage have limited the sustainability of the table grape industry. A novel plant growth regulator, 24-epibrassinolide (EBR), exhibits an essential regulatory function in plant life cycles, especially in its unique mechanism against various environmental stresses. We treated ‘Lihongbao’ grapes with exogenous EBR (0.2 μM), brassinazole (BRZ, 10 μM), EBR + BRZ (0.2 μM +10 μM), and deionized water (CK). We investigated the effect of exogenous EBR on ‘Lihongbao’ grape seedlings under low-temperature stress (4°C) at different periods (0 h, 12 h, 24 h, 48 h, and 96 h). We explored physiological mitigation mechanisms of exogenous EBR in grape seedlings with low-temperature injury by observing the impacts of EBR treatment on the physical and biochemical indices such as phenotypes and anatomical structures, photosynthetic characteristics, chlorophyll fluorescence parameters, antioxidant systems, and osmoregulatory substances. Exogenous EBR had an inhibitory effect on cold stress in grape seedlings at different treatment periods compared with the CK group. Based on plant phenotype and anatomical structure, the leaves of the grape seedlings treated with exogenous EBR showed no signs of water loss or wilting. At 96 h under low-temperature stress, the lower epidermal thickness (LET), palisade tissue thickness (PT), palisade-to-sea ratio (P/S), and blade structural compactness (CTR) of the exogenous EBR-treated grape leaves were significantly reduced by 6.71%, 19.59%, 14.52%, and 11.65% compared with the CK group, respectively. Chlorophyll a (Chl a), chlorophyll b (Chl b), total chlorophyll (Chl total), carotenoids (carotenoid), transpiration rate (Tr), and stomatal conductance (Gs) contents of exogenous EBR-treated grape leaves were significantly upregulated by 30.24%, 48.52%, 39.75%, 34.67%, 704.66%, and 277.27%, respectively. The intercellular CO_2_ concentration (Ci) and non-photosynthetic burst coefficient (NPQ) of exogenous EBR-treated grape leaves were significantly downregulated by 16.29% and 25.83%, respectively. Glutathione (GSH) contents of exogenous EBR-treated grape leaves were significantly upregulated by 33.63%, superoxide dismutase (SOD), peroxidase (POD), and ascorbate peroxidase (APX) activities of exogenous EBR-treated grape leaves were significantly increased by 42.70%, 27.60%, and 28.64%, respectively. However, hydrogen peroxide (H_2_O_2_), superoxide anion (O_2_·^−^), and malondialdehyde (MDA) contents of exogenous EBR-treated grape leaves were reduced by 29.88%, 23.66%, and 47.96%, respectively, and significantly. Catalase (CAT) activity of exogenous EBR-treated grape leaves significantly increased by 15.03%. Soluble sugar and free proline contents increased by 5.29% and 19.44%, respectively, and significantly. Exogenous EBR could effectively alleviate growth inhibition caused by regulating the antioxidant system indices in grape seedlings under cold temperature. The results offer a theoretical basis for enhancing grape cold tolerance.

## 1 Introduction

Grapes (*Vitis* spp.) occupy an important position in the global fruit industry and have long been ranked among the top six fruits of the world due to their unique growth habits and rich nutritional value. Grapes are primarily cultivated in temperate and subtropical areas of the Northern Hemisphere. Their fruits have considerable economic value due to excellent quality loved by most consumers (Yang et al., 2024). Grape, with its rich bioactive substances, constitutes a unique nutritional barrier. The polyphenols contained therein can not only remove free radicals from the body and slow down the aging process but also show amazing potential in the fight against cancer and the inhibition of inflammatory reactions (Ji et al., 2019). China is one of the major origins and largest number of grapes. It is also one of the richest genetic resources worldwide (Pan et al., 2024). China has the third largest area of planted vineyards, is the eighth largest wine consumer, and is the first table grape producer worldwide (International Organization of Vine and Wine, 2024a). After a long period of expansion from 2000 to 2015, vineyard growth in China slowed down with approximately 756,000 ha in 2023, which represents a decrease of 0.3% compared with 2022 (International Organisation of Vine and Wine, 2024b). The ‘Lihongbao’ grape used in this experiment was validated and named in March 2010 by the Shanxi Provincial Variety Validation Committee. The ‘Lihongbao’ grape is a new medium-maturing non-nuclear variety bred from ‘Guibao’×’Centennial Seedless’, which is specific to the Institute of Fruit Tree Research of Shanxi Academy of Agricultural Sciences and has high research value.

Low-temperature stress is a key factor leading to a reduction in production and injury of fruit trees (Jalili et al., 2023). Grape cultivation faces new challenges and threats, including various stresses that severely impact on fruit production. Cold stress poses a significant environmental challenge, directly influencing plant survival, geographic distribution, vegetative development, and agricultural productivity (Dey et al., 2023). Among challenges facing plants in nature, low temperature, an abiotic stressor, has a particularly significant impact on their life activities. When the temperature plummets, the physiological and biochemical processes in plants will be seriously interfered with, thus affecting their normal growth, and ultimately leading to the irreversible consequence of crop yield reduction (Xu et al., 2022b; Askari-Khorasgani et al., 2019; Kou et al., 2018). As a horticultural plant of high economic value, grapes have relatively limited resistance to low temperatures, a characteristic that deserves special attention in agricultural production. It could cause changes in the dynamic balance between cells under low-temperature stress. Plants undergo a huge change wherein coordinated mechanisms evolve, conferring low-temperature resistance (Xu et al., 2022b). Low-temperature stress causes inhibition of various biological processes in plants and results in changes in their physiological mechanisms of action. The cold attack causes the plant’s photosynthesis system to be severely damaged, and the stomata on the surface of the leaves have to be closed tightly. The stress response causes the leaves to take on a sickly yellow color. This physiological damage not only is reflected in the leaves but also directly affects the growth of plant. This leads to a decrease in biomass and triggers an array of physiological and biochemical responses, which disrupt the metabolic systems of plants and ultimately cause an obvious decline in productivity (Takeuchi et al., 2022; Gao et al., 2024). This has a direct impact on the fluidity of cell membranes and the functionality of enzymes and also influences cellular osmotic processes and responses to reactive oxygen species (ROS) in an indirect manner (Dey et al., 2023). The osmotic pressure imbalance and oxidative stress caused by this stress are intertwined to form a puzzle. This double whammy has an incalculable impact on the normal metabolic processes of plant cells (Dreyer and Dietz, 2018). When ice crystals form in cold environments, a complex physiological process is initiated in the plant: an osmotic pressure imbalance triggers a chain reaction that leads to the accumulation of soluble sugars and proline, in large quantities in the cells. The metabolites by participating in the intricate metabolic pathways ultimately serve as essential protectors of cells during critical damage moments (Zhang et al., 2019). Nevertheless, the persistent buildup of ROS in cold conditions gives rise to oxidative stress. In living organisms, excessive accumulation of ROS will cause irreversible damage to the organism, so that lipid molecules suffer from peroxidation damage, nucleic acid structure is destroyed, and protein conformation changes. This chain reaction of damage will eventually trigger a series of metabolic disorders (Qin et al., 2023). Therefore, it is crucial to implement measures to mitigate the harm caused by low-temperature stress in grapevine seedlings.

In the complex regulatory network in plants, brassinosteroids (BRs) play an indispensable role. Its ability to form a dynamically balanced signaling network in the plant enables the plant to maintain normal development and appropriate stress responses in the face of unfavorable environments (Nie et al., 2019). 24-Epibrassinolide (EBR) refers to a class of polyhydroxysteroidal phytohormones that serve as BR analogs (Heidari et al., 2021). They are essential for plant development. BRs as a class of endogenous steroid hormones widely found in plants, which not only regulate the growth and differentiation of plant cells but also play a pivotal role in photosynthesis. Amazingly, BRs also give plants the ability to withstand various adverse environmental factors, so that they show amazing resilience (Yang et al., 2019). EBR regulates diverse plant growth cycles and is crucial for stress defense (Hu et al., 2022; Seleiman et al., 2023). Brassinazole (BRZ) is a specific inhibitor of BR synthesis, involved in the BR signaling pathway in plants (Mumtaz et al., 2022). Exogenous EBR mitigates the negative effects of stress on plants by influencing stomatal conductance, enhancing root system vitality, and optimizing root morphological characteristics. Through its regulatory role, EBR promotes efficient gas exchange by modulating stomatal aperture, ensuring adequate CO_2_ supply under stress conditions. Concurrently, it strengthens root activity by improving water and nutrient uptake efficiency. These interconnected mechanisms collectively contribute to the alleviation of stress-induced suppression (Emamverdian et al., 2022). EBR enhances antioxidant levels and reduces malondialdehyde (MDA) content to mitigate lipid peroxidation harm to cell membranes (Mansoreh et al., 2021). EBR has also been used to modulate the activity of plant antioxidant enzyme regulators, such as superoxide dismutase (SOD), peroxidase (POD), catalase (CAT), and ascorbate peroxidase (APX), and to promote the accumulation of non-enzymatic systems, such as glutathione (GSH) and ascorbic acid (AsA) contents (Ma et al., 2015). Exogenous EBR has been used to regulate the metabolic homeostasis of plants under cold stress, to induce the accumulation of osmoregulatory substances. It effectively removes reactive oxygen such as hydrogen peroxide (H_2_O_2_) and superoxide anion (O_2_·^−^) from the cells and downregulates plant cell membrane permeability. Obviously, it enhances recovery by promoting the ROS system, osmotic regulation, and hormonal metabolism during extreme situations (Zeng et al., 2022).

However, limited information exists on how exogenous EBR mitigates grapevine seedlings damage under cold stress. We conducted a detailed examination of how exogenous EBR influences the physiological processes, photosynthetic efficiency, and antioxidant defense mechanisms in grape seedlings exposed to low temperature. By analyzing the interplay between these factors, this study tries to elucidate the regulatory roles of EBR in mitigating the adverse effects under cold stress in grapevine growth.

## 2 Materials and methods

### 2.1 Plant materials

The study was conducted from March to October 2022–2024 at the Horticulture Experimental Center of Shanxi Agricultural University. The grape branches for the test were the Eurasian species of table grapes ‘Lihongbao’. The grapes were harvested from the National Grape Germplasm Resource Nursery of Shanxi Agricultural University, Taigu, Shanxi, China (112°32′ E, 37°23′ N, mean annual temperature 9.8°C). The cuttings of the winter sand-stored ‘Lihongbao’ grape branches were rooted on 25 March 2022 and planted in moist fine sand for rooting. After 2 months, the plants were transplanted into pots containing raw soil, perlite, and humus (2:1:1, v/v/v). The seedlings were grown in a greenhouse for 9 weeks and irrigated once a week with tap water and 1/2 Hoagland nutrient solution. After the grapes had grown to 8–10 functional leaves, 60 uniformly sized, uniformly growing grape seedlings were selected for subsequent trials. A total of 60 grape seedlings were grouped into four according to a completely randomized design. There were 15 seedlings within each group, with five representing one biological replicate for each of the five low-temperature stress durations, replicated three times in total.

Grape seedlings were used for four treatments with deionized water only (as control, CK), 0.2 μM EBR (EBR), 10 μM BRZ (BRZ), and 0.2 μM EBR and 10 μM BRZ (EBR + BRZ). In particular, the above reagents in four treatments were dissolved with 98% ethanol at a content of 0.1% (v/v) and then diluted with deionized water, and it was also used as an unfolding agent with Tween-20 at a content of 0.1% (v/v). Three of the four groups were sprayed with reagents on the front and back of the leaves until they dripped. The seedlings were transferred to a light incubator (16°C/4°C day/night, 55% relative humidity, 10,000 1× light intensity, 14/10 h light/dark photoperiods) for low-temperature stress following 6 days of continuous treatment. The seedling morphology was observed at five time points (0 h, 12 h, 24 h, 48 h, and 96 h) from each treatment group. Seedlings were selected for photosynthetic fluorescence parameters by counting the third to fifth fully expanded functional leaves from bottom to top. Samples were immediately transferred to liquid nitrogen and stored at −80°C for further analysis.

### 2.2 Observation of morphological structure in grape seedling leaves

Plant growth was recorded by capturing photographs with a digital Canon camera at five time points of low-temperature stress from three seedlings randomly selected from the treatment groups.

### 2.3 Observations on the structure in grape seedling leaves

In this experiment, small square slices of 1 × 1 cm in the center of leaves, adjacent to the main vein for each treatment, were selected and rapidly placed in formalin–acetic acid–alcohol (FAA) fixative for fixation. This was followed by paraffin sectioning, ethanol gradient dehydration, xylene transparency, paraffin embedding, toluidine blue staining, and sealing with Canada gum. The sections were then placed under an ordinary light microscope to select the sections for observation and photographed using an Olympus DP71 (Japan) microimaging system. Simultaneously, the system’s tool was used to measure upper epidermal thickness (UET), lower epidermal thickness (LET), fence tissue thickness (PT), sponge tissue thickness (ST), and leaf thickness (LT) for five indices. The blade organization and structure parameters such as palisade-to-sea ratio (P/S), blade structural compactness (CTR), and leaf tissue structure sparseness (SR) were calculated according to the following equations:


P/S=PT÷ST

CTR(%)=(PT÷LT)×100%

SR(%)=(ST÷LT)×100%

### 2.4 Photosynthetic pigment content in grape seedling leaves

The photosynthetic pigment contents of the leaves were measured using direct ethanol extraction (Anwar et al., 2018). 1 g of samples was placed in a 25 mL stoppered test tube, and 10 mL of 95% ethanol was added for thorough mixing. The tubes were left in the dark for 48 h to ensure that leaves lost their green color and whitened, then shaken well every 12 h. The filtrate was used as the extract, and the absorbance values were determined at 470 nm, 665 nm, and 649 nm.

### 2.5 Gas exchange parameters in grape seedling leaves

Photosynthetic gas exchange parameters of leaves at the same position in seedlings were measured using a LI-6800 Portable Photosynthesis System (LI-COR, Nebraska, USA) from 9:00 to 11:00 am. The main measured parameters were transpiration rate (Tr), photosynthetic rate (Pn), intercellular CO_2_ concentration (Ci), and stomatal conductance (Gs). The specific parameters of the photosynthesizer for the index measurements were set to the leaf chamber light intensity of 500 μmol·m^−2^·s^−1^, the airway flow rate of 300 μmol·s^−1^, and the leaf chamber mixing fan speed of 5,000 r·min^−1^.

### 2.6 Chlorophyll fluorescence parameters in grape seedling leaves

A portable fluorometer (FluorPen FP110, Czech Republic) was used to determine the functional leaves of each fully expanded treatment at the same leaf position at night, and the plants were adequately dark-adapted for at least 2 h prior to measurement. The maximum photochemical efficiency of Photosystem II (Fv/Fm), the actual photochemical efficiency of Photosystem II (φPSII), the non-photosynthetic burst coefficient (NPQ), and the photochemical burst coefficient (qP) were determined.

### 2.7 Active oxygen content in grape seedling leaves

(1) O_2_·^−^ content

O_2_·^−^ content was determined using the method described by Zhao et al. (2016). 1 g of samples was weighed and added to 50 mM phosphate buffer (PBS) in batches and was fully ground, homogenized, and centrifuged for 15 min. Then, 2 mL of the supernatant of the extract and 2 mL of p-aminobenzenesulfonic acid (17 mM) was added sequentially with 2 mL of α-naphthylamine (7 mM). Next, the tubes were incubated in a 30°C water bath for 30 min and cooled to 20°C, and then the absorbance values at 530 nm were determined.

(2) H_2_O_2_ content

The H_2_O_2_ content was measured with a kit (Beijing Solepol Technology Company, Ltd.). 0.1 g of samples was added to the extract in batches to be ground and homogenized in an ice bath. They were added to 1 mL of precooled acetone for overnight maceration. This was followed by centrifugation at freezing temperature for 20 min, and the supernatant was aspirated to determine the H_2_O_2_ content.

### 2.8 Antioxidant content in grape seedling leaves

(1) AsA content

AsA content was determined using the method described by Liang et al. (2018). 1 g of samples and 6 mL of 5% trichloroacetic acid (TCA) solution were ground into a homogenate in an ice bath, added into tubes. The test tubes were subjected to freeze centrifugation for 15 min, and the supernatant was collected. Next, 1 mL of the supernatant was added to a test tube, followed by 1 mL of 5% TCA solution, 0.5 mL of 0.4% phosphoric acid–ethanol solution, 1 mL of anhydrous ethanol, 0.5 mL 0.03% FeCl_3_–ethanol solution, and BP–ethanol solution (1 mL). The mixture was shaken well and then placed in a water bath at 30°C for 60 min. The absorbance was measured at 525 nm.

(2) GSH content

GSH content was determined using the method described by Liang et al. (2018). 1 g of samples and 10 mL of precooled 5% TCA (containing 5 mM Na_2_EDTA) solution were ground into a homogenate in an ice bath, added into tubes. The tubes were then cryocentrifuged for 15 min, and the supernatant was collected. Next, 1 mL of supernatant, 1 mL of 0.1 mM PBS solution, and 0.5 mL of 4 mM DTNB solution were added with sufficient shaking. The absorbance value was measured at 412 nm.

### 2.9 Antioxidant enzyme activities in grape seedling leaves

0.6 g of samples and 6 mL of PBS (50 mM, pH 7.8) were ground into a homogenate in an ice bath, added into tubes. This was followed by centrifugation at freezing for 30 min, and the supernatant was collected for enzyme activity assays (Ozlem, 2022).

(1) SOD activity

The SOD activity was determined using the nitrogen blue tetrazolium method (Zeng et al., 2022). 0.1 mL of supernatant and 3 mL of reaction solution were added. The reaction solution was prepared with 130 mM methionine, 20 μM riboflavin, 750 μM nitro blue tetrazolium (NBT), and 100 μM EDTA with 50 mM PBS (pH 7.8). The reaction solution was placed under 10,000 1× culture light for 10 min, and the absorbance was measured at 560 nm under light-avoidance conditions.

(2) POD activity

The POD activity was determined using the guaiacol method (Zeng et al., 2022). A mixture of 3 mL containing 76 μL of guaiacol, 112 μL of H_2_O_2_, and 200 mL of PBS (pH 7.0) was mixed with 200 μL of enzyme solution. The CK tube was used with the mixture and 200 μL of phosphate buffer, and the timing was started immediately. Absorbance was measured at 470 nm.

(3) CAT activity

The CAT activity was determined using the hydrogen peroxide decomposition method (Zeng et al., 2022). The prepared enzyme solution 200 μL was placed in a cuvette. 3 mL of reaction solution containing 100 mL PBS (pH 7.0) and 154.6 μL H_2_O_2_ were added. The absorbance was measured at 240 nm.

(4) APX activity

The APX activity was determined using the hydrogen peroxide decomposition method (Zeng et al., 2022). 100 μL of enzyme solution and 2.8 mL of 50 mM PBS (pH 7.0) containing 0.1 mM EDTA and 0.5 mM AsA were added and then mixed thoroughly. Finally, 100 μL of 20 mM H_2_O_2_ was added to start the reaction. Absorbance was measured at 290 nm.

### 2.10 The content of oxidative stress indexes in grape seedling leaves

MDA content was determined using the colorimetric method of thiobarbituric acid (Zhong et al., 2020). 0.5 g of samples and 0.5% (w/v) TCA were added in the ice bath and thoroughly ground, and then cryocentrifuged for 20 min. The supernatant and 0.5% TCA were placed in a tube, shaken well, boiled in a water bath for 30 min, and finally centrifuged for 10 min for twice. The supernatant was determined at 450 nm, 532 nm, and 600 nm.

### 2.11 Content of osmoregulatory substances in grape seedling leaves

(1) Free proline content

Free proline content was determined using the ninhydrin colorimetric method (Wang et al., 2022). 0.2 g of samples and 5 mL of 3% sulfosalicylic acid were placed in tubes and covered with a boiling water bath for 15 min. The samples were centrifuged for 15 min for twice. Subsequently, 2 mL of the second supernatant was added to a centrifuge tube with 2 mL glacial acetic acid and 3 mL acidic ninhydrin, allowed to react for 30 min in a boiling water bath. Next, 5 mL of toluene was added, shaken well, and left to stand for 2–3 h away from light. Absorbance was measured at 520 nm.

(2) Soluble sugar content

Soluble sugar content was determined using the anthrone-ethyl acetate method (Li et al., 2022). 0.05 g of samples and 10 mL of deionized water were added, shaken well, and boiled in a water bath for 30 min. The tubes were then centrifuged for 10 min. Next, 0.5 mL of the supernatant, 1.5 mL of deionized water, and ethyl anthrone acetate (0.5 mL) were added into the tubes. 5 mL of concentrated H_2_SO_4_ was slowly added, and the reaction was carried out in a boiling water bath for 1 min with rapid shaking. Absorbance was measured at 620 nm.

### 2.12 Statistical analysis

Statistical analysis of the data was performed using Microsoft Office 2016. SPSS 26.0 was used for correlation analysis (p < 0.05), cluster analysis, and principal component analysis. All the data results were expressed as mean ± SE of three replicates. OriginPro 2021 and RStudio were used to plot the graphs, and Photoshop 2023 was used for drawing.

## 3 Results

### 3.1 Effects of exogenous EBR in grape seedling growth under low-temperature stress

**Figure 1:**
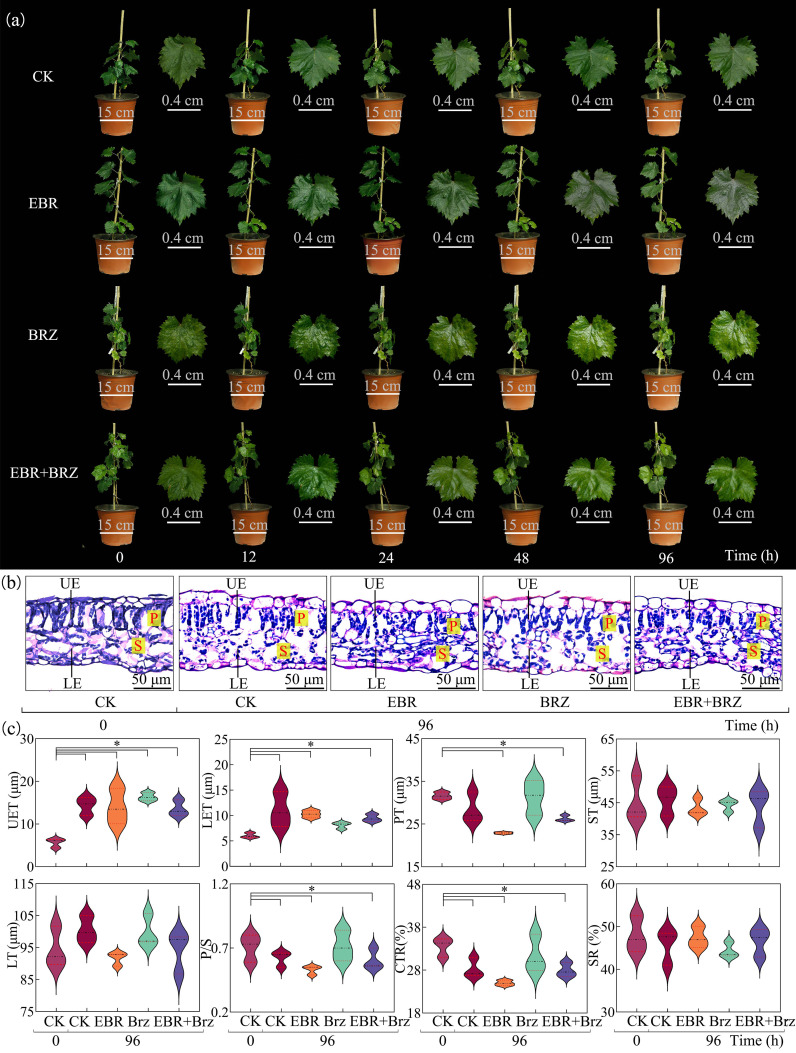
Different treatments including CK, EBR, BRZ, and EBR + BRZ on the phenotypic characteristics and leaf microstructure under low-temperature stress. **(A)** Phenotypic characteristics. **(B)** Microstructures. UE, upper epidermis; LE, lower epidermis; P, palisade tissues; S, spongy tissues. **(C)** Anatomical structure indexes at 96 h. The * in the figure indicate significant differences between treatments (P≤0.05).

Leaves of all the grape seedlings exhibited different degrees of damage over time, as shown in **Figure 1A**. Exogenous EBR treatment minimized low-temperature injury in grape seedlings compared with the CK group, and at 0 h, there were no substantial differences in all treatments. After 12 h of low-temperature stress, the leaves in the CK were slightly water-lost and drooped. The leaf margins were curled. However, no notable changes in leaf morphology were observed in the EBR, BRZ, and EBR + BRZ groups. After 24 h, the leaves in the BRZ lost some water and drooped. The edges of the leaves were slightly curled. The CK group showed significant water loss in the leaves of grape seedlings, with prominent curling of the leaf margins and low-temperature damage. After 48 h, the water loss drooping of leaves with curled edges gradually recovered in the EBR group. In the CK group of grapevine seedlings, water loss and drooping leaves were obvious; the edges of the leaves showed fewer yellowing spots, and the plants were more severely damaged. After 96 h, there were no obvious signs of damage to the leaves in the EBR group. The leaves observed in the EBR + BRZ group showed minimal signs of dehydration, accompanied by a subtle degree of wilting. The leaves in the CK group showed significant water loss and wilting, accompanied by leaf abscission. The leaves showed different degrees of damage with the prolongation of cold stress, and exogenous EBR helped alleviate the damage to the leaves.

### 3.2 Effects of exogenous EBR on microstructure in grape seedling leaves under low-temperature stress

**Figure 1B** shows that the fenestrated and spongy tissues of the leaves were closely arranged under normal growth conditions, and the upper and lower epidermal cells were morphologically intact. There was a different degree of damage to the grape plants in each treatment group following low-temperature treatment. Exogenous EBR mitigated the damage to the morphological structure of leaves after 96 h. Specific manifestations showed that the cellular morphology was more complete, and the fenestrated and spongy tissues were more tightly arranged. The anatomical structures of the leaves in the EBR group could not be distinguished. However, the main manifestations were broken upper and lower epidermal cells of the leaves, spongy tissue, and poorly differentiated fenestrated tissue in the CK, BRZ, and EBR + BRZ groups. Exogenous BRZ mitigated damage to the morphological structure of leaves but was relatively ineffective, and BRZ inhibited the effects of EBR. Exogenous EBR protected the structure of leaves under low-temperature stress. In **Figure 1C**, the stress caused a decrease in seedling leaf UET, LET, PT, ST, LT, P/S, and CTR, with a significant decrease in PT and CTR. The exogenously sprayed EBR group showed a significant reduction of 19.59% in PT and 11.65% in CTR compared with the CK group after 96 h. Exogenous EBR treatment significantly alleviated the damage to leaf anatomical structures from low temperature.

### 3.3 Effects of exogenous EBR on photosynthetic pigments in grape seedling leaves under low-temperature stress

**Figure 2:**
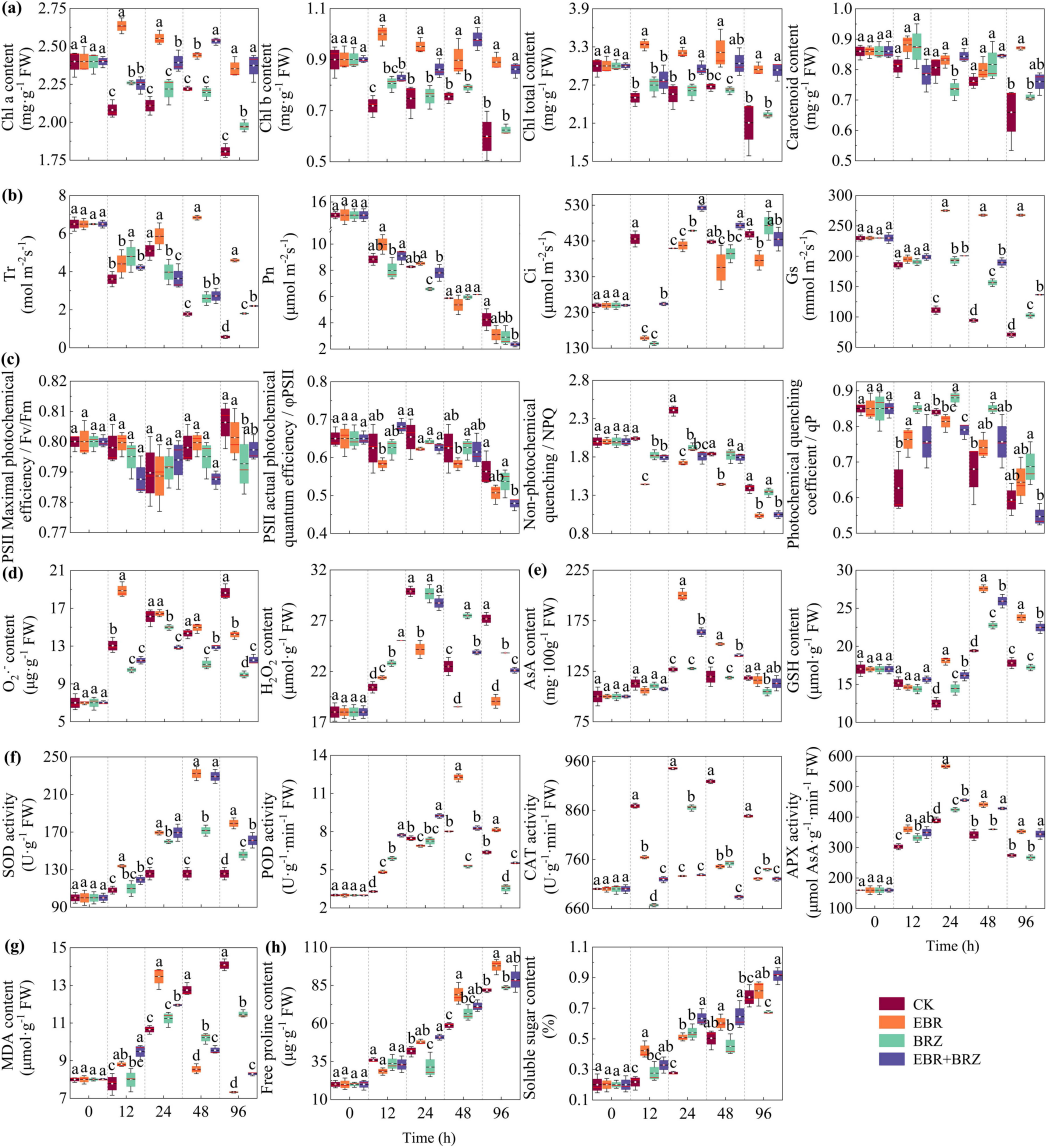
Different treatments including CK, EBR, BRZ, and EBR + BRZ on physiological indexes of grape leaves under low-temperature stress. Different lowercase letters in the figure indicate significant differences between treatments (P ≤ 0.05). **(A)** Photosynthetic pigments (Chl a, Chl b, Chl total, and carotenoid). **(B)** Gas exchange parameters (Tr, Pn, Ci, and Gs). **(C)** Chlorophyll fluorescence parameters (Fv/Fm, φPSII, NPQ, and qP). **(D)** Content of O_2_·^−^ and H_2_O_2_. **(E)** Content of antioxidants (AsA and GSH). **(F)** Antioxidant enzyme activities (SOD, POD, CAT, and APX). **(G)** Content of oxidative stress indexes (MDA). **(H)** Content of osmoregulatory substances (free proline and soluble sugar).

Photosynthetic pigments are crucial in plant physiology, with their concentration in leaves directly indicating the plant’s photosynthetic activity. The Chl a, Chl b, Chl total, and carotenoid contents under different treatments were determined at five sampling periods. **Figure 2A** shows that the response of different treatments and different pigment contents was not the same. There was an overall decreasing trend, followed by an increasing trend, and then decreasing Chl a, Chl b, and Chl total contents in the leaves of the CK, BRZ, and EBR + BRZ groups. This was characterized by a rapid decline within 12 h, a steady increase from 12 h to 48 h, and a rapid decline from 48 h to 96 h. The Chl a content significantly increased by 9.27% in the BRZ group at 96 h compared with CK. The contents of Chl a, Chl b, and Chl total significantly increased by 31.39%, 43.96%, and 39.44%, respectively, in the EBR + BRZ group at 96 h compared with CK. However, the trends in Chl a, Chl b, and total Chl content in EBR group were different from those in the CK group. It showed a rapidly increasing trend, reaching a peak after 12 h, followed by a gradual decrease. The Chl a, Chl b, and Chl total contents of the exogenous EBR treatment grapevine seedling leaves were higher than in the CK group. The contents of Chl a, Chl b, and Chl total significantly increased by 30.24%, 48.51%, and 39.75%, respectively, at 96 h compared with CK. The carotenoid content in CK, BRZ, and EBR + BRZ grape seedlings steadily decreased during 96 h. However, the trend in carotenoid content in the EBR group was different from the CK group, with an overall decreasing trend followed by an increasing trend. Exogenous EBR treatment leaves had a higher carotenoid content than the CK during 48 h, but the difference was insignificant. A significant difference was observed at 96 h compared with the CK group, which increased by 34.66%. The photosynthetic pigment contents decreased with prolonged low-temperature stress. Exogenous EBR powerfully mitigated the harm caused by low-temperature stress in grape seedlings and increased the content of photosynthetic pigments, while it enhanced the photosynthetic intensity. However, combined application of EBR and BRZ reduced photosynthesis.

### 3.4 Effects of exogenous EBR on gas exchange parameters in grape seedling leaves under low-temperature stress

Photosynthesis not only dominates the conversion and accumulation of energy in the plant but also determines whether or not the entire plant can grow and thrive. Tr, Pn, Ci, and Gs were determined for each treatment plant. As shown in **Figure 2B**, the response to the cold stress was not the same for the different treatments and gas exchange parameters. The trend of Tr in the grape seedlings in the four treatment groups was the same from 0 h to 24 h, with a decreasing and then increasing trend. The changes occurred after 24 h, and Tr in grape seedlings of the CK, BRZ, and EBR + BRZ groups showed a gradual decrease with prolonged stress. However, Tr in the grape seedlings of the EBR group showed an increasing trend. It reached its highest value at 48 h and then gradually decreased to reach a significant level compared with CK. The four treatment groups showed a decreasing trend in Pn. The Pn values of EBR and CK groups did not reach significant differences after 96 h, but the Pn values in the BRZ and EBR + BRZ treatment groups were obviously different from the CK group. The overall trend of Ci in grapevine seedlings in the four treatment groups was upward, and the change in Ci in grapevine seedlings in the CK group stabilized after a significant increase at 12 h. Compared with the CK group, the Ci in the EBR, BRZ, and EBR + BRZ groups first decreased obviously at 12 h, then increased at 24 h, and then showed a relatively stable trend after reaching the highest value. The Gs in the CK, BRZ, and EBR + BRZ groups showed a significant decrease with the prolongation. However, the trend of Gs changes in EBR treatment differed from the CK group. After 12 h, it decreased rapidly and then increased significantly with the extension of the cold time to reach the highest value and then stabilize. The Tr, Pn, and Gs of grape seedling leaves decreased, and Ci increased with prolonged stress. Exogenous EBR alleviated the decrease in Tr and Gs and the increase in Ci but did not significantly alleviate the changes in Pn. Exogenous BRZ helped to alleviate the decrease in Tr and Gs but promoted the decline of Pn and did not significantly alleviate the changes in Ci under low-temperature stress.

### 3.5 Effects of exogenous EBR on chlorophyll fluorescence parameters in grape seedling leaves under low-temperature stress

The chlorophyll fluorescence parameter could reflect the degree of harm and the level of cold tolerance. The Fv/Fm, φPSII, NPQ, and qP parameters were determined for each treatment plant at different low-temperature times. **Figure 2C** shows that the different treatments and chlorophyll fluorescence parameters did not respond equally to cold stress. The change trends in Fv/Fm, φPSII, NPQ, and qP parameters in grape seedlings was the same in the four treatment groups. Fv/Fm decreased and then increased with the prolongation of stress. The Fv/Fm in BRZ treated was significantly different from the CK group after 96 h, and BRZ effectively suppressed the increase in Fv/Fm values. The trends of PSII, NPQ, and qP decreased with the prolongation of stress. Among them, the use of BRZ alone did not significantly affect φPSII, NPQ, and qP after 96 h, but coupled with EBR, it significantly promoted the reduction. Meanwhile, the EBR group only significantly promoted NPQ after 96 h and did not form an obvious difference in the changes of Fv/Fm, φPSII, and qP values. Fv/Fm in grape seedling leaves did not change significantly with the extension, and φPSII, NPQ, and qP decreased with the extension of stress time. The exogenous EBR helped to promote the rise in Fv/Fm and the decline of φPSII and NPQ in the leaves and alleviated the decline in qP. Exogenous EBR coupled with BRZ improved the chlorophyll fluorescence parameters of leaves.

### 3.6 Effects of exogenous EBR on ROS contents in leaves in grape seedlings under low-temperature stress

ROS are metabolic by-products in plant chloroplasts, mitochondria, and peroxisomes and have a dual function in adversity stress with a dose effect. The contents of O_2_·^−^ and H_2_O_2_ were determined under each treatment plant. **Figure 2D** shows that the O_2_·^−^ content of the CK group had an overall increasing trend except for 48 h. However, the trend of O_2_·^−^ content changes in EBR, BRZ, and EBR + BRZ treatment grape seedlings was different from that of the CK, with a significant increase at 12 h. This was followed by a notable decreasing trend with the prolongation of stress. Among them, the O_2_·^−^ content in the leaves of EBR-, BRZ-, and EBR + BRZ-treated grape seedlings was significantly reduced by 23.66%, 46.50%, and 38.16%, respectively, at 96 h compared with CK. The H_2_O_2_ content of the CK group increased steadily before 24 h and then decreased and increased from 24 h to 96 h, with an overall increasing trend. However, the H_2_O_2_ content in the EBR, BRZ, and EBR + BRZ groups tended to increase and then decrease, reaching the highest value at 24 h, following by a gradual decline. Subsequently, the H_2_O_2_ content was lower than the CK group at 24 h, 48 h, and 96 h, with significant reductions of 19.13%, 17.65%, and 29.88%, respectively, in the EBR group. The H_2_O_2_ and O_2_·^−^ content in grape seedling leaves increased with the duration of stress. Exogenous EBR in the late stage of low-temperature stress could effectively alleviate the low-temperature damage suffered by grape plants and inhibit the rise in O_2_·^−^ and H_2_O_2_ contents.

### 3.7 Effects of exogenous EBR on antioxidant contents in grape seedling leaves under low-temperature stress

Antioxidants, such as AsA and GSH, regulate the redox state of plant cells and balance ROS levels. In this experiment, the AsA and GSH contents of treated plants were determined at different times. **Figure 2E** shows that the AsA content in the CK group gradually increased, peaked after 24 h, and then gradually decreased. The change in the AsA content of grape seedling leaves in the EBR, BRZ, and EBR + BRZ groups showed a trend that was the same as the CK group. The AsA content of leaves peaked at 24 h, was notably higher than CK, and then showed a decreasing trend with prolonged stress time. AsA content of grape seedling leaves at 24 h significantly increased by 57.63% and 29.07% in the EBR and EBR + BRZ groups, respectively. At 48 h, it significantly increased by 27.08% and 17.48% in the EBR and EBR + BRZ groups, respectively. EBR effectively increased AsA content in the leaves of the plants, and BRZ exhibited some inhibitory effects. The trend in the GSH content in grape plants in the four treatment groups remained the same throughout the experiment. The GSH content decreased in the early stage of stress, followed by a gradual increase and then a decrease in GSH content. GSH peaks appeared in the four treatment groups after 48 h. The GSH content of EBR-treated was substantially greater than in the CK group. At 24 h, 48 h, and 96 h, the GSH content significantly increased by 45.36%, 41.99%, and 33.63%, respectively, in the EBR group compared with CK. The AsA and GSH content of grape plants increased with prolonged stress. Exogenous EBR enhanced the content of AsA and GSH in grape plants and mitigated the low-temperature harm to grape plants.

### 3.8 Effects of exogenous EBR on antioxidant enzyme activities in grape seedling leaves under low-temperature stress

Antioxidant enzymes are crucial components of the plant antioxidant enzyme system, which work together to scavenge excess ROS in the defense against cold stress. EBR-treated effectively mitigated low-temperature damage in grape plants, increased the contents of antioxidant substances, and enhanced the antioxidant capacities of grape to tolerate cold stress. Therefore, we conducted SOD, POD, CAT, and APX analyses for each treatment group at different cold times. As shown in **Figure 2F**, SOD activity in CK grape seedlings increased and then stabilized, reaching a peak after 24 h. Meanwhile, the SOD activity in EBR, BRZ, and EBR + BRZ groups showed a trend of increasing and then decreasing, reaching a peak at 48 h. SOD activity was obviously higher in the EBR and EBR + BRZ groups than in the CK group. Compared with CK at 24 h, 48 h, and 96 h, the SOD activity of grapevine seedling leaves significantly increased by 34.80%, 84.84%, and 42.70% in the EBR group; 19.21%, 36.53%, and 15.98% in the BRZ group; and 34.79%, 82.48%, and 28.34% in the EBR + BRZ group, respectively, during the middle and late stages of stress. The trends in POD, CAT, and APX activities in all groups first increased and then decreased. The POD activity of grape seedling leaves peaked at 48 h and increased significantly by 52.97% in EBR compared with CK. The CAT and APX activities peaked after 24 h and were significantly reduced by 23.18% and 45.96% in EBR compared with CK. At the end of the stress period, the activities of SOD, POD, and APX were markedly higher, and the activity of CAT was lower in the EBR group than in the CK group. Meanwhile, BRZ and EBR + BRZ treatments either enhanced or suppressed antioxidant enzyme activities in grape seedlings, but the effects were not as prominent as those of the EBR treatment. The enzymatic antioxidant activity of grapevine seedling leaves increased with stress. Exogenous EBR significantly elevated the activities of SOD, POD, and APX, in grape seedling leaves and inhibited the activity of CAT.

### 3.9 Effects of exogenous EBR on the content of oxidative stress indexes in grape seedling leaves under low-temperature stress

As shown in **Figure 2G**, the MDA changes in the CK group showed an upward trend, and the MDA content of the EBR, BRZ, and EBR + BRZ groups showed an increasing and then a decreasing trend. MDA content significantly decreased after reaching its maximum value after 24 h. At 48 h and 96 h, EBR treatment had the strongest inhibitory effect on the MDA content, which was reduced by 32.95% and 47.96%, respectively, compared with the CK group. An indicator of oxidative stress of grapevine seedlings, the MDA content gradually increased with the prolongation of cold stress. Exogenous EBR treatment markedly reduced the accumulation of MDA content exposed to low-temperature stress. This intervention significantly suppressed the rise in MDA levels.

### 3.10 Effects of exogenous EBR on the contents of osmotic mediators in grape seedling leaves under low-temperature stress

Plants can synthesize osmoregulatory substances to regulate osmotic pressure and change water potential to improve plant resistance. The free proline and soluble sugar contents of the treated plants were determined. **Figure 2H** shows that the trend of free proline and soluble sugars in the four treatment groups has an increasing trend. The free proline content in grapevine seedlings was markedly increased by 34.45% and 19.44% at 48 h and 96 h, respectively, in the EBR group compared with CK. Soluble sugar content of grape seedling leaves increased by 84.72%, 19.71%, and 5.29% at 24 h, 48 h, and 96 h, respectively, compared with CK. With the prolongation of cold stress, EBR-treated obviously increased soluble sugar content compared with CK. However, EBR + BRZ treatment had a better promotion effect. The soluble sugar content of grape seedlings increased by 129.19%, 27.83%, and 18.14% at 24 h, 48 h, and 96 h in the EBR + BRZ group compared with the CK group. The free proline and soluble sugar contents were significantly greater in the EBR group than CK after 96 h. The content of free proline and soluble sugar in grape seedlings increased with prolonged cold stress. When grapevine seedlings were subjected to exogenous EBR treatment and subsequently exposed to low-temperature stress, there was a notable rise in the levels of free proline and soluble sugars. This unique physiological regulation mechanism has shown its indispensable value in alleviating the damage caused by low temperature.

### 3.11 Evaluation of exogenous EBR on leaf indexes in grape seedling leaves under low-temperature stress

**Figure 3:**
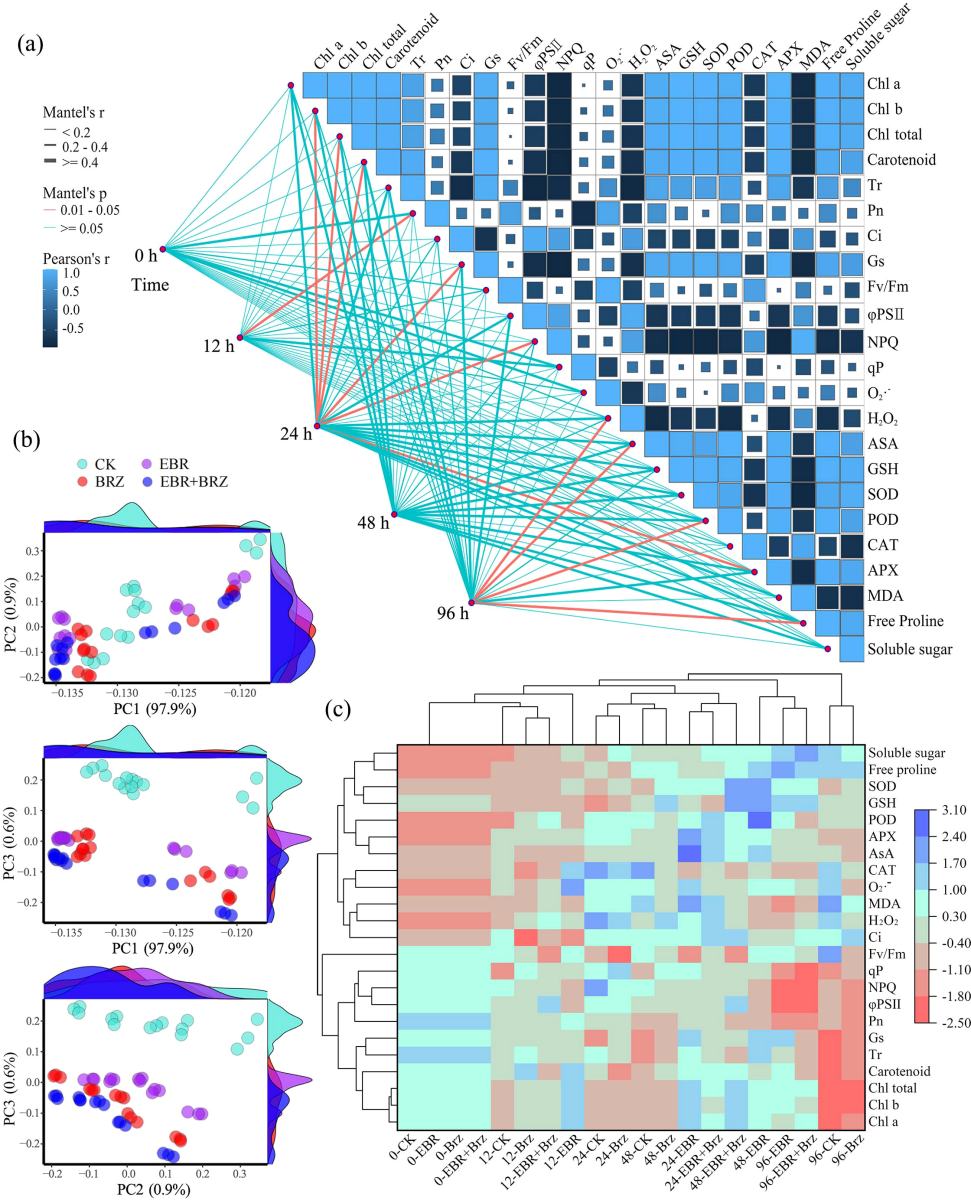
Different treatments including CK, EBR, BRZ, and EBR + BRZ on the physiological indexes under low-temperature stress. **(A)** Correlation analysis. **(B)** PCA analysis. **(C)** Cluster analysis.

As shown in **Figure 3A**, the 23 physiological indices were correlated with five matrix types grouped by low-temperature stress time. Pn notably correlated with low-temperature stress at 0 h. Chl b, Chl total, carotenoids, Gs, NPQ, and APX were significantly correlated with stress at 12 h. H_2_O_2_, AsA, POD, and free proline were significantly correlated with stress at 96 h. However, no significant correlation was observed between 0 h and 48 h and the physical and chemical indices. Low-temperature stress treatments significantly affected the leaf photosynthetic pigments, photosynthetic gas exchange parameters, and chlorophyll fluorescence parameters of grapevine seedlings after 48 h. The results of this study are summarized as follows. Low-temperature stress treatment for 48 h to 96 h significantly affected the indices of ROS, antioxidant substances, antioxidant enzyme activity, and osmotic adjustment substances.

As shown in **Figure 3B**, the physiological and biochemical indices of grape cuttings after exogenous EBR spray treatment were subjected to principal component analysis (PCA). Three principal components with the highest eigenvalues, PC1 (97.9%), PC2 (0.9%), and PC3 (0.6%), were extracted. The samples treated with EBR and CK exhibited significant sample separation at different low-temperature treatment times.

As shown in **Figure 3C**, based on the standardized data of physiological indexes of EBR sprayed grapevine seedlings under different cold stress time treatments, the seedling leaf samples of five low-temperature periods were clustered by clustering heatmap analysis to study their stoichiometric characteristics. The results showed that the 20 treated samples could be categorized into three groups. The first group of treatment samples was CK and BRZ after 96 h. The second group of treatment samples was EBR for 48 h and 96 h and EBR + BRZ for 96 h. The third group of treatment samples was CK for 0 h, 12 h, 24 h, and 48 h; EBR for 0 h, 12 h, and 24 h; BRZ for 0 h, 12 h, 24 h, and 48 h; and EBR + BRZ for 0 h, 12 h, 24 h, and 48 h. The cluster analysis plot showed that the 23 physiological indicator samples could be categorized into two groups. The first group of indicator samples included soluble sugars, free proline, SOD, GSH, POD, APX, AsA, CAT, O_2_·^−^, MDA, H_2_O_2_, and Ci. The second group of indicator samples comprised Fv/Fm, qP, NPQ, φPSII, Pn, Gs, Tr, carotenoid, Chl total, Chl b, and Chl a. Exogenous spraying of EBR effectively counteracted the low-temperature stress experienced by grapevine seedlings and was dominated by the modulation of osmoregulatory substances and antioxidant system indices in grape leaves.

## 4 Discussion

**Figure 4:**
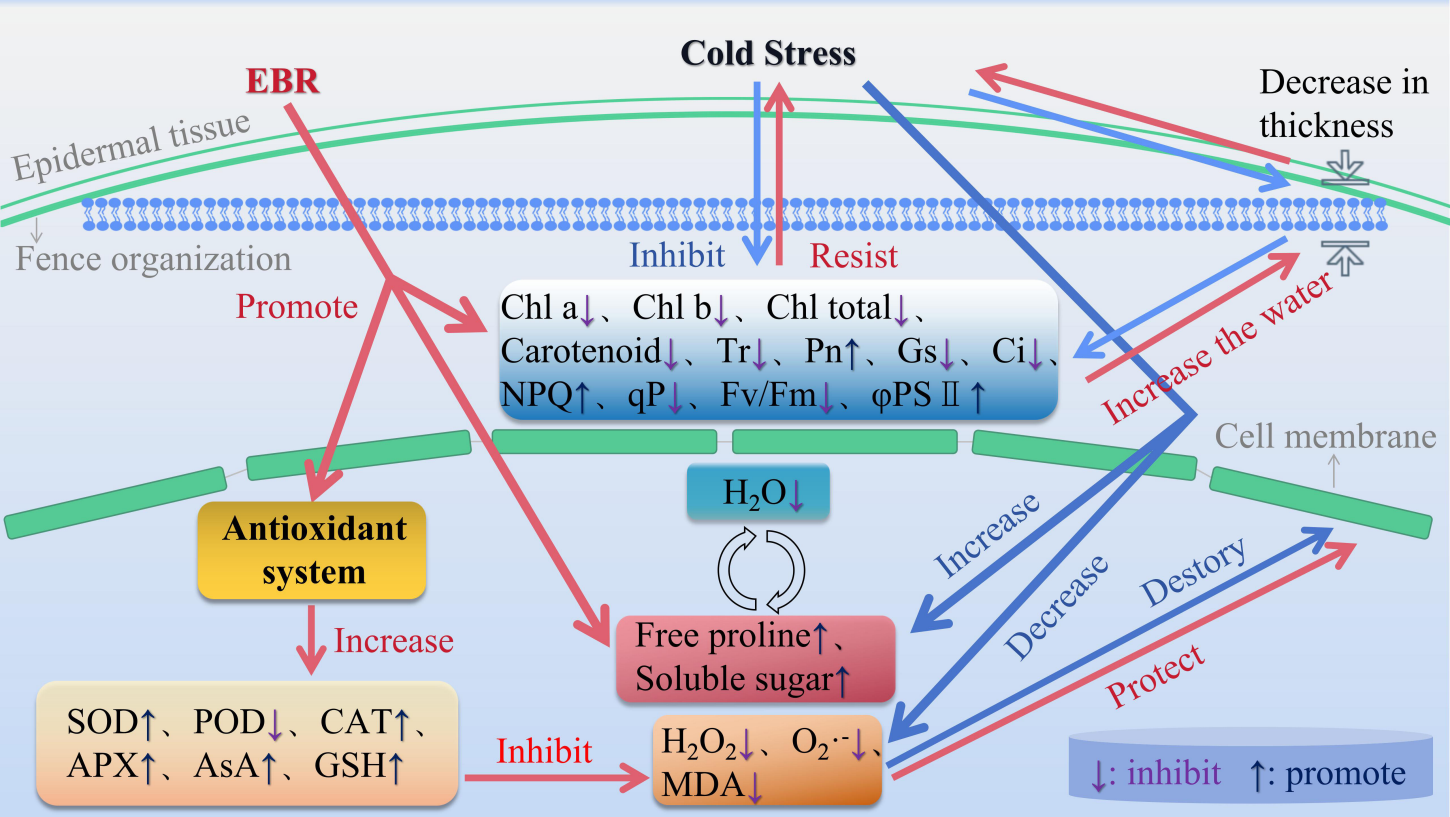
Schematic diagram of the physiological response mechanism of exogenous EBR to 96 h of cold stress in grape seedlings.

With increasing cold stress duration, the normal life activities of grape seedlings were inhibited, and their growth and development stagnated. Based on the changes in each index and the results of the comprehensive analysis, that is, correlation, principal component, and cluster analyses, the comprehensive ability of grape seedlings to resist cold stress after exogenous EBR treatment was greater than that of the CK group. As shown in **Figure 4**, exogenous EBR application significantly mitigated cold stress damage in grape seedlings, offering potential for widespread use in viticulture practices.

### 4.1 Effects of exogenous EBR on the external morphology and anatomical structure of grape seedling leaves

The harm caused by low temperatures in plants is reflected in both phenotypic features and anatomical structures and physiological and biochemical indices. Phenotypic traits of plants experiencing low-temperature stress usually include symptoms such as plant wilting and leaf necrosis (Lu et al., 2019). Morphological changes in grapevine seedlings were investigated in four treatments, CK, EBR, BRZ, and EBR + BRZ, at 0 h, 12 h, 24 h, 48 h, and 96 h, respectively. There was a tendency for wilting and water loss in the leaves of seedlings in the CK group at 12 h, 24 h, 48 h, and 96 h, which were significantly damaged by low-temperature stress. Although no significant damage was observed in EBR, more significant damage was observed in BRZ. However, the EBR, BRZ, and EBR + BRZ groups were more resistant to cold stress than CK. In the physiological process of plants subjected to cold stress, the application EBR showed a significant protective effect. This discovery opened up new research ideas to improve the cold tolerance of plants. EBR regulated a series of complex pathways in plants to build up a physiological barrier against cold attack. It was worth pondering that the establishment of this protective mechanism is far more subtle than we have imagined, which not only is reflected in the improvement of the apparent morphology but also involves the maintenance of the stability of the cell membrane and the dynamic balance of osmotic regulation at a deeper level. The scientific findings revealed by the experimental study of Chen et al. (2019) coincide with this phenomenon, with its insights into the EBR processing mechanism showing significant mitigating effects in response to cold stress damage. The tightly arrange fenestrated tissues of plants exposed to cold stress could effectively maintain water retention in grapes (Lu et al., 2019). After the leaves were subjected to stress, the leaves fenestrated and spongy tissues were loosely arranged, and LT, PT, and ST were reduced. In the research field of oil tea seedling growth and development, Xu et al. (2022a) confirmed the scientific validity of this viewpoint through a series of experimental design. Exogenous EBR treatment resulted in a more complete leaf cell morphology, a tighter arrangement of fenestrated tissues, and no significant reduction in LT, ST, or SR. In contrast, the fenestrated tissue was loosely arranged in the CK group after 96 h. Exogenous spraying of EBR protected the leaf structure of grape seedlings and alleviated the stressful effects of adversity on grapes. In the study of Wang et al. (2022), the effects of EBR on the anatomical structure of oil tea leaves under low-temperature stress showed a similarity, a finding that not only corroborates the conclusions of the present study but also provides a new way of thinking for in-depth exploration of phytohormone regulatory mechanisms.

### 4.2 Effects of exogenous EBR on photosynthetic and fluorescence properties of grape seedling leaves

When plants suffer from low-temperature stress, photosynthesis, a central mechanism of plant life activity, inevitably gets disrupted (Guan et al., 2023; Lee et al., 2021). Accumulation of photosynthetic pigments in plants showed a significant attenuation under the stress of cold environments. It was worthwhile to pay attention to the fact that, by applying exogenous EBR, this unavoidable attenuation process seemed to be alleviated to a certain extent, so that the rate of loss of photosynthetic pigments showed a relatively moderate trend, which were consistent with Li (2020) and Anwar et al. (2018). The importance of photosynthesis as a fundamental way for plants to obtain energy cannot be overstated. It was worth pondering that when the ambient temperature is persistently low, this core system of plant survival will be inhibited, which might trigger a series of chain reactions affecting growth of plant (Yudina et al., 2020). In grape seedlings, cold stress resulted in a significant decrease in Pn, Tr, and Gs, and a significant increase in Ci. This diminished the leaves’ photosynthetic traits, resulting in reduced chloroplast activity, a finding that coincides with the experimental data of Yang et al. (2019). In contrast, exogenous EBR and EBR + BRZ pretreatments restored the levels of Tr, Pn, and Gs and reduced Ci levels. This showed that damage to the photosynthetic system of grape seedlings triggered by low temperatures was significantly alleviated by exogenous EBR and that the blockage of photoinhibition and the increase in photosynthetic capacity could lead to an increase in CO_2_ uptake by the plants. These experimental data coincide with the findings reached by Chen et al. (2019). The electron transport chain of photosynthesis showed an irreversible blockage during low-temperature stress. This altered physiological mechanism upset the dynamic equilibrium between the absorption of light energy by the photosynthesis system and the energy consumption during metabolism, which was completely disrupted. It was worth pondering that this imbalance triggers a series of chain reactions: excessive excitation energy accumulated in the photosynthetic system inevitably induced photoinhibition of PSII (Jin et al., 2022). The values of Fv/Fm, qP, NPQ, and φPSII decreased with the increase in stress time. Cold stress inhibited the conversion efficiency of PSII primary light energy and the potential photosynthetic activity of PSII in grapevine seedling leaves. The chlorophyll fluorescence properties of grapevine seedling leaves responded strongly to cold stress. The decreases in Fv/Fm, NPQ, qP, and PSII in the leaves treated with EBR and EBR + BRZ were markedly lower than CK-treated leaves. EBR showed a significant protective effect under cold stress, which was negatively correlated with the degree of photosystem damage, when exploring the mechanism of the effect of exogenous EBR on the grapevine photosystem, aligning with the results of Stachurska et al. (2022) and Sadura and Janeczko (2022).

### 4.3 Effects of exogenous EBR on the antioxidant system of grape seedling leaves

Plant cells maintain a delicate balance of reactive oxygen species, an equilibrium that is particularly vulnerable to cold stress. When the cold comes, the homeostasis inside the cell is ruthlessly broken, and O_2_·^−^ and H_2_O_2_, two substances, begin to accumulate. It is worth pondering that these reactive oxygen molecules, which are cellular metabolites under normal physiological conditions, not only are the key bioindicators of oxidative stress in plants but also harness their strengths against various threats to life (Singh et al., 2024). The accumulation of MDA, an important product of lipid peroxidation in cell membranes during plant stress, has always been regarded as a key indicator of the degree of harm to membrane systems. By analyzing the content of MDA, the oxidative damage suffered by cell membranes can be evaluated, and this approach is of irreplaceable value in the study of plant adversity physiology (Zhao et al., 2023). In this study, low-temperature stress caused a notable rise in O_2_·^−^ and H_2_O_2_ levels, alongside enhanced activities of SOD, POD, CAT, and APX, and an increase in AsA, GSH, and MDA levels. The homeostatic balance between ROS scavenging and accumulation was disrupted, and the ROS attack resulted in peroxidative damage to the cytoplasmic membrane. This aligns with the results reported by Amin et al. (2022). In contrast, the MDA content under EBR was obviously lower than CK, and the O_2_·^−^ and H_2_O_2_ contents were lower than CK. Meanwhile, the activities of SOD, POD, CAT, and APX were notably higher than in the CK group, together with AsA and GSH. Exogenous EBR and EBR + BRZ treatments alleviated the extent of oxidative harm to the cell membrane of grapevine seedling leaves. Excess ROS were efficiently scavenged in the plants, whereas membrane lipid peroxidation was mitigated. These findings align with Chen et al. (2019) and Yang et al. (2019).

### 4.4 Effects of exogenous EBR on osmoregulatory substances of grape seedling leaves

Stress regulatory responses are found in plants when they encounter an adverse environment, in which osmoregulation is a crucial pathway (Khanna et al., 2023). Low-temperature environments cause water condensation in plant cell tissues, dehydration of the protoplasm, denaturation of proteins, and irreversible mechanical damage (Kumari et al., 2022). The osmoregulatory functions of plants are stimulated by stress, and free proline and soluble sugars are important osmoregulators (Altaf et al., 2024). Low-temperature stress resulted in a substantial increase in the free proline and soluble sugar contents in grape. This cold environmental challenge caused varying extents of cell membrane injury, accompanied by membrane lipid peroxidation, which disrupted the structural and functional integrity of the cellular membrane system. Such observations align with the research outcomes reported by Guo et al. (2020) and Karimi (2020), underscoring the susceptibility of plant cellular structures to oxidative damage under cold stress conditions. In contrast, the free proline and soluble sugar contents of exogenous EBR and EBR+BRZ-treated seedling leaves were enhanced. The application of EBR mitigated the detrimental effects induced by low-temperature stress while exerting a notable regulatory influence on the concentrations of osmoregulatory substances. These results align closely with the observations documented by Yang et al. (2019) and Heidari et al. (2021), further substantiating the hypothesis regarding EBR in mitigating abiotic stress through physiological adjustments.

## 5 Conclusions

Spraying exogenous EBR under cold stress protected the structure of grapevine seedling leaves, inhibited photosynthesis and chlorophyll fluorescence, increased the accumulation of photosynthetic pigments, regulated the accumulation of antioxidant enzymes and non-enzymatic antioxidants, and promoted the accumulation of osmotically regulated substances and the catabolism of MDA. The enhancement of the cold resistance in grapevine seedling leaves significantly strengthens their ability to withstand extreme stress, reducing the extent of damage caused by such environmental conditions. In the process of exploring the cold resistance mechanism of grapevine, the application value of EBR has been impressively verified. This discovery provides a new way of thinking for grape growing under a low-temperature environment. The practical significance of this research far exceeds the theoretical level, opening up new possibilities for the development of the grape industry in cold regions, although the specific regulatory mechanisms of EBR under cold stress have not been fully elucidated in grape. In future, the mechanism of exogenous EBR remains to be deeply analyzed. By intertwining EBR with transcriptomics and metabolomics, we may be able to unravel the regulation of EBR in grapevine under extreme stress conditions. This idea is expected to provide theoretical support for the practical application of grapevine cultivation.

## Data Availability

The original contributions presented in the study are included in the article/supplementary material. Further inquiries can be directed to the corresponding author.
